# A short bout of low to moderate aerobic exercise influences cognitive performance in healthy adults based on task type, timing, and individual factors

**DOI:** 10.3389/fspor.2025.1712545

**Published:** 2026-02-04

**Authors:** Cornelia Herbert

**Affiliations:** Applied Emotion and Motivation Psychology, Institute of Psychology and Education, Ulm University, Ulm, Germany

**Keywords:** acute bout of aerobic exercise, arousal, cognitive domain specificity, cognitive performance, healthy adults, heart rate variability, memory, neurovisceral integration

## Abstract

**Background:**

Many young adults experience stress and poor mental health, which can negatively impact cognitive performance and overall well-being. Short aerobic exercise sessions may help enhance cognitive processing in healthy individuals, but some questions remain**.**

**Aim/methods:**

This study examined how a brief 16-minute aerobic exercise session, involving cycling at low to moderate intensity, affects verbal memory, fluency, and cognitive switching before, during, or after exercise across two testing sessions—one with exercise and one without—in 19 healthy young adults (both men and women). Verbal learning and memory were assessed before and after exercise, whereas verbal fluency and cognitive switching were assessed during the exercise. Performance was compared with the control condition of “no exercise” using a crossover design to investigate order-dependent selective effects of testing time and context (i.e., whether exercise occurred during the first or second session). Mean heart rate (mean HR), vagally mediated heart rate variability (HRV), and self-reported physical activity levels were also measured to control for physiological arousal, habitual physical activity, and self-regulation (HRV).

**Results:**

Cognitive performance varied across tasks. The results suggest order-dependent selective effects of the testing time. Correlations between individual HRV at rest or habitual physical activity, mean heart rate during exercise, and performance were found in exploratory analyses.

**Conclusion:**

Short bouts of low-to-moderate aerobic exercise modulate cognitive processing in healthy adults. However, this depends on the order-dependent selective effects of exercise timing and whether the tasks are new or familiar. The findings underscore the importance of these factors in determining when and how short aerobic exercise sessions affect cognitive performance in healthy individuals. The results are limited by factors such as a small sample size. Recommendations are provided to address these limitations and explore ways to improve them.

## 1 Introduction

Sedentarism, insufficient physical activity, self-reported stress, and poor mental health have significantly increased among young adults (1, 2). These trends can negatively impact cognitive performance and well-being and raise the risk of non-communicable diseases (NCDs) (1–4). As a result, healthy adults have become a key target group for health promotion initiatives (5).

Regular physical activity and regular aerobic exercise are successful health promotion interventions emphasized in international health guidelines for all age groups (6–8). Due to their positive impact on health and cognitive function (9–11), research has also investigated whether acute bouts of aerobic exercise can enhance cognitive processing and performance. In healthy adults, several types of physical exercise have been examined, including aerobic and strength/resistance exercise (10–19). Moreover, the effects of an acute bout of aerobic exercise have been extensively studied, particularly in healthy adults [for reviews, meta-analyses, or summaries, see (12–19)].

Overall, previous reviews and meta-analytic studies have shown promising results, with small yet positive effect sizes (12, 13), particularly for moderate-intensity aerobic exercise compared to higher or lower intensities. These moderate-intensity findings partly support the proposed relationship between the arousal triggered by a single bout of aerobic exercise and its impact on cognitive processing, as described by the Yerkes-Dodson law [e.g. (18, 20),]. The law suggests an inverted U-shaped relationship between the intensity of the physical/physiological arousal triggered by the aerobic exercise and the cognitive demands of a task. Therefore, single bouts of aerobic exercise should enhance cognitive processing and performance during mental tasks, provided the exercise is of moderate intensity. However, some reviews suggest that acute bouts of low-intensity aerobic exercise can provide benefits identical to those of moderate-intensity aerobic exercise in healthy individuals. This discrepancy may indicate potential biases in previous research investigating exercise intensity (13, 15). Additionally, recent findings highlight the positive effects of high-intensity exercise bouts (21, 22).

The effects of exercise intensity on cognitive processing cannot be understood in isolation from other factors. Additional factors must be considered, such as the duration of the exercise, the specific cognitive processing involved, and the timing of the exercise and cognitive performance testing. There is evidence that for regular exercise, the frequency, duration, and intensity of weekly repetitions are essential for achieving health benefits according to international guidelines, including cognitive effects of exercise (6–8). However, there is currently insufficient evidence to determine the optimal duration of a single session of aerobic exercise and its impact on cognitive processing; for a summary and discussion, see e.g. (12), and (13, 15).

While some previous reviews and meta-analyses suggest that approximately 20 min of moderate-intensity exercise is most beneficial, this recommendation may apply only to specific types of tasks [for an overview, (12–15)]. Additionally, the effectiveness of a single session of aerobic exercise on cognitive processing may depend on whether the exercise and cognitive processing occur simultaneously or in sequence, and whether the exercise is performed at a steady state or until exhaustion (21, 22).

Engaging in acute bouts of aerobic exercise can negatively affect cognitive performance when performed simultaneously in a dual-task context (14). As a result, cognitive performance may decline during the exercise compared to before or during the recovery period afterward. Conversely, performance on the same or similar tasks might remain unchanged or improve when comparing results before and after an exercise session (17, 21, 22). For example, in dual-task situations, aerobic exercise and verbal, cognitive, and motor tasks may interfere with each other because they depend on shared neural resources, especially for breathing, cognitive, motor, and executive function control (for details, see Discussion). Combining exercise with cognitive tasks that demand substantial respiratory, cognitive, and motor effort can reduce processing capacity, potentially affecting performance of the physical and cognitive tasks (for details, see Discussion).

Therefore, and importantly, which type of cognitive task and whether the accuracy or the speed of mental processing is investigated can account for considerable variance in the findings (16, 23–25): studies have shown that after a single session of moderate intensity aerobic exercise, cognitive functions—particularly cognitive inhibition, which is controlled by the prefrontal cortex (especially the dorsolateral prefrontal cortex)—improve. For cognitive functions associated with the hippocampal memory system of the temporal lobe, the impact of timing, exercise intensity, and duration appears less clear (26). Moreover, for memory processing, the timing of the acute bout of exercise—before, during, or after the learning, consolidation, and retrieval phases of memory —seems to be critical (27). Furthermore, it is important to distinguish between the effects of exercise on working memory (26, 27) and on long-term memory, as well as its impact on declarative or episodic memory (14, 26).

In summary, previous studies have shown that several factors can influence the effect of a single session of aerobic exercise on cognitive processing in healthy adults. Key factors include the timing of the exercise, the type of cognitive tasks, and the cognitive performance measures chosen. Furthermore, the duration and the intensity of the aerobic exercise are important, particularly when examining short-duration exercises with varying intensities below or above moderate.

Finally, individual and inter-individual differences are often discussed in the literature as important factors influencing the effectiveness of an acute bout of aerobic exercise on cognitive performance. Aside from age and gender, individual differences in physical activity and cardiovascular fitness may influence the effects of acute aerobic exercise on physical, mental, and cognitive health (23, 28). Moreover, some previous studies indicated differences between individuals who score high and low on cognitive functions including functions required for verbal, and logical reasoning (23).

### 1.1 Aim of the present study

As pointed out above and in detail in many previous studies and reviews summarized above, acute bouts of aerobic exercise can boost cognitive performance in healthy adults. Nevertheless, several questions remain about the conditions and factors that could contribute to this enhancement, particularly during a single short-duration exercise session and during lower-intensity aerobic exercise bouts. Therefore, with the rise in sedentary behavior, insufficient daily physical activity, and the growing number of self-reported mental health issues among healthy adults, it is essential to explore these factors further. It is important to explore, a) how an acute bout of aerobic exercise of low to moderate intensity and duration modulates cognitive performance in healthy adults when b) different cognitive tasks are considered, c) performance is assessed pre-post or during the exercise session, and d) compared across different cognitive testing sessions (with or without exercise). Doing so might help develop health promotion recommendations, for example, on how to incorporate physical activity breaks in the daily routines of healthy adults (29, 30). In line with this effort, this study aimed to examine the impact of order-dependent selective effects of testing time, and context on cognitive performance, specifically concerning when and how short aerobic exercise sessions—such as a single 16-minute low to moderate cycling session—affect cognition in healthy adults across two different cognitive tasks and testing sessions. Performance under these conditions was compared with the control condition of “no exercise” and across two testing situations: one in which tasks were novel and another in which they were familiar. Two standardized cognitive tasks were selected: a word-fluency task and a verbal memory task to evaluate how cycling influences cognitive functions, including memory and cognitive flexibility. The chosen tasks are associated with several cognitive functions, including learning, memory consolidation and retrieval, cognitive inhibition, cognitive flexibility and cognitive switching, and other executive functions such as working memory and cognitive control, which are governed by prefrontal brain networks and temporal lobe memory systems (see the Method section below). Additionally, the study assessed whether the effects of exercise on cognition vary based on participants' self-reported levels of regular physical and cardiovascular activity.

Methodologically, the design allows the exploration of the hypothesis of cognitive domain specificity (14, 31), i.e., whether exercise-cognition effects are task-specific or task-general, including measures of accuracy and speed of mental processing. It allows for the examination of the role of order-dependent selective effects of exercise timing across different days of cognitive testing, taking into account serial-order effects of the tasks including task novelty and familiarity, as well as individual factors. Both cognitive and psychophysiological outcome measures were explored. The latter included investigating changes in mean heart rate to estimate the physiological arousal elicited by the acute bout of exercise and the cognitive tasks, and analyzing participants' resting heart rate variability (HRV).

Heart rate variability (HRV), especially its vagally mediated components, can indicate the degree of parasympathetic activation at rest and the strength of its control by higher-order brain centers of the so-called CAN (Central-Autonomic-Network) (32). Therefore, resting heart rate variability (HRV) may serve as a proxy for the participants' degree of central autonomic self-regulation. It may provide a theoretically interesting parameter for the study of exercise-cognition interactions in healthy adults (33–37), as HRV has been associated with cardiovascular and cognitive performance related to self-regulatory control of attention, memory, and cognitive functions such as cognitive flexibility and control. Increases in physiological arousal elicited by physical and mental activity typically increase sympathetic activity of the autonomic nervous system. Therefore, this study examines whether and how variations in HRV at rest, as an index of parasympathetic cardiac regulation and a theoretical and empirically reliable marker of PFC (prefrontal cortex) functioning and self-regulatory control, are related to task and exercise performance across experimental conditions.

In summary, the study explores the following research questions:

Does a 16-minute bout of low to moderate cycling exercise increase the accuracy of verbal memory performance before and after the exercise compared to the performance without exercise?
Does the same bout of exercise improve cognitive fluency and cognitive switching during the exercise beyond the performance without exercise?
Are the exercise effects modulated differently by task novelty and familiarity across the two testing sessions with and without exercise?
What is the role of the exercise-induced physiological arousal in cognitive performance effects?
Is cognitive performance related to vagally mediated HRV as an indicator of an individual's self-regulatory capacity?
Is there a relationship between the self-reported physical activity of the participants and their cognitive performance in the exercise condition?

## 2 Methods

### 2.1 Participants

A total of 20 participants (10 women and 10 men) were healthy adults who took part in the study. Participants were recruited through local email lists, advertisements, and flyers distributed on the university campus. To be eligible, individuals had to be at least 18 years old, native German speakers, and free from any history of neurological, somatic, cardiovascular, or mental disorders, as well as from medication taken for these conditions. Additional exclusion criteria included pregnancy, current injuries, and any viral or infectious diseases at the time of testing. The intake of caffeine, nicotine, or alcohol was controlled. The inclusion and exclusion criteria, including substance intake, were assessed through anamnestic questions and habitual physical activity was assessed using the German version of the Physical Activity Questionnaire (38).

Before participating in the study, the participants were debriefed about the purpose of the study and its procedures. Participation was voluntary; individuals could opt out at any time without providing reasons or facing any consequences. All participants provided written informed consent before their involvement. Although participants were not compensated individually, they could enter a raffle for a shopping voucher or receive credit points if they were psychology students. All methods and procedures described in this manuscript followed the relevant guidelines and regulations. The study and study design were approved by the local ethics committee of Ulm University (https://www.uni-ulm.de/einrichtungen/ethikkommission-der-universitaet-ulm/).

### 2.2 Procedure and study design

Upon arrival and after providing written informed consent, the participants completed a series of anamnestic and socio-demographic questions. They were asked about their current mood, habitual physical activity levels, and weekly exercise routines (see Table 1). Participants were invited to the two testing sessions on two different days. Each participant was scheduled for the same time on the two consecutive days. The interval between the two laboratory days was kept constant for all participants, ranging from four to six days, to maintain control over the cognitive retention interval across the two testing sessions.

**Table 1:** Sociodemographic data, current mood (positive and negative affect), and habitual/regular physical activity assessed via standardized questionnaires (see text for details). PA: Physical activity (minutes). For details, see Methods and Results.

Sociodemographic data: age and habitual physical activity	mean	Standard deviation (SD)
mean age (women and men)	24.32	2.83
PA: vigorous at work	0.00	0.00
PA: moderate at work	92.63	204.40
PA: transport	239.74	212.46
PA: vigorous-recreation/leisure	211.05	202.16
PA: moderate-recreation/leisure	143.68	122.87
PA: total per day	98.16	74.38
Positive Affect	27.79	5.82
Negative Affect	12.11	2.29

#### 2.2.1 Testing sessions—study protocol

**Figure 1:**
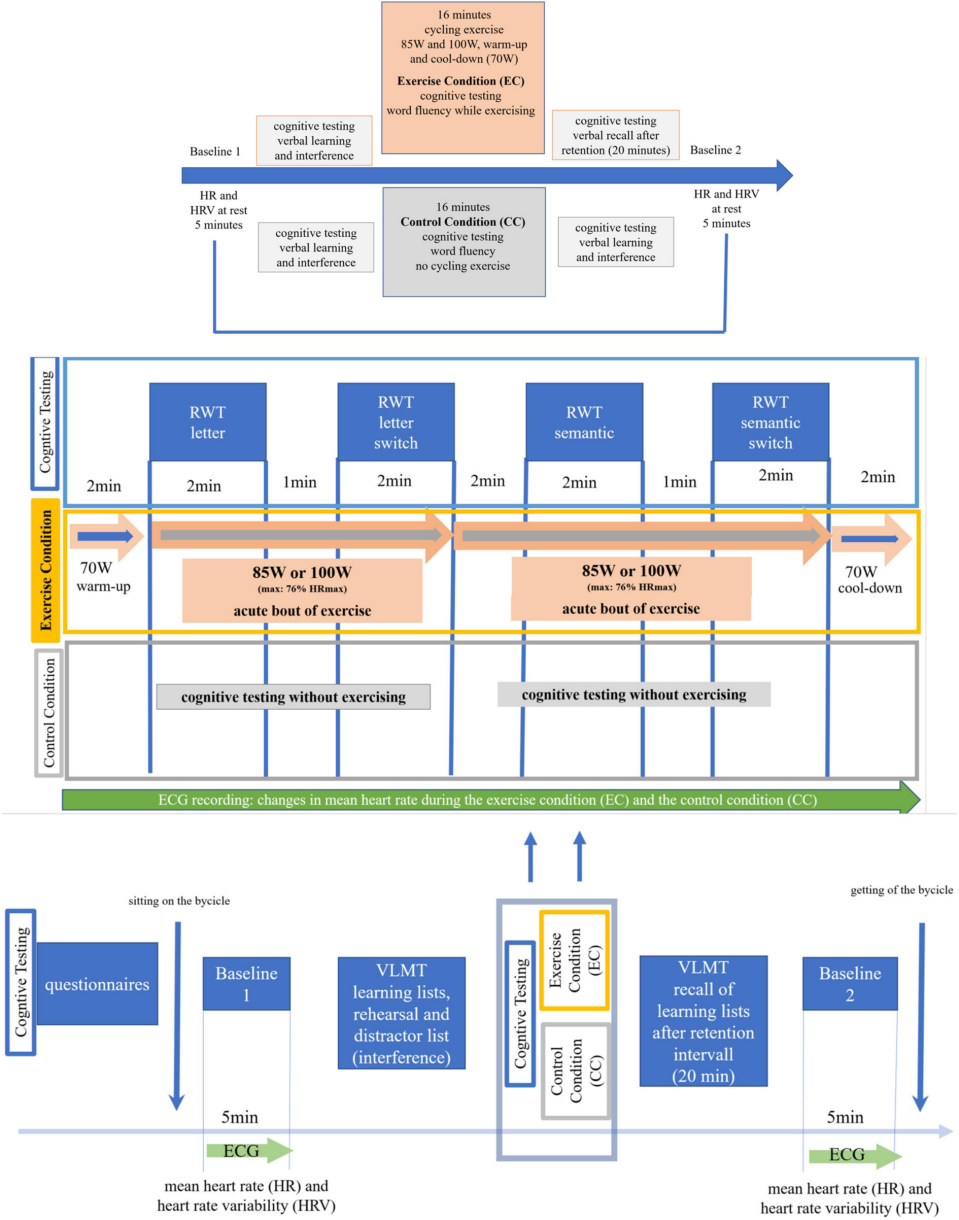
Overview of the experimental study design. For a detailed description, please see the text.

For each participant, the two laboratory sessions comprised the exercise condition (EC) on one day and the control condition of no physical exercise (CC) on the other day. In both experimental conditions (EC and CC), the participants underwent two baseline measurements and conducted two cognitive tasks. In the physical exercise condition (EC), the baseline measurements were taken before and after the exercise and the cognitive testing. Similarly, the control condition (CC) included the two baseline measurements (pre- and post) and the three blocks of cognitive testing, which, however, were conducted without physical exercise. Therefore, the procedure was identical for both experimental conditions (EC and CC), except that no acute bout of exercise was carried out in the CC (see Figure 1). The participants were randomly assigned to one of two options: in option A, day 1 was EC and day 2 was CC; in option B, day 1 was CC and day 2 was EC. In the first testing session, the tasks were novel to the participants; in the second testing session, the participants were already familiar with the tasks. The order of participation in the exercise condition (EC) and control condition (CC) was counterbalanced among participants, ensuring an equal distribution of self-reported gender to minimize gender biases.

On each of the two laboratory days and in each experimental condition (EC or CC), the participants began with the 5-minute baseline measurement (baseline 1), during which their cardiac activity was recorded. To control for differences in cardiac activity due to body position, the participants were asked in both experimental conditions (EC and CC) to sit still, relaxed, and quietly on the cycling ergometer in an upright position, with their hands on their upper legs and their legs and feet comfortably resting without pedaling. The second baseline measurement of 5 min was recorded after the intervention, assessing cardiac activity at rest following recovery from cognitive testing (CC) or combined cognitive and exercise testing (EC).

At the end of the first laboratory day, participants were scheduled for their second session, which included the EC or CC conditions, depending on their initial assignment to option A or option B (see above). For a detailed summary and overview of the study design, see Figure 1.

#### 2.2.2 Acute bout of aerobic exercise of low-to-moderate intensity

The acute bout of aerobic exercise consisted of cycling. The cycling exercise was performed on a professional ergometer for health and endurance exercise (the Ergobike Premium 8i; Daum Electronics). The exercise session lasted 16 min. It included 2 min of warm-up and 2 min of cool-down. The Ergobike software allows the control of exercise intensity via exercise load (WATT). Therefore, the exercise load (WATT) was set at low intensity (50%–63% of maximum heart rate) or moderate intensity (64%–76% of maximum heart rate). Maximum heart rate (HRmax) was estimated using the age-predicted HRmax equation (i.e., 220-age), which is well established for healthy young adults. This resulted in an estimated HRmax of 191.2 beats per minute (bpm) as an average reference value for a mean age of 24 years in the study sample. A mean heart rate of 122.4–145.3 beats per minute for a mean age of 24 years corresponded to exercise of moderate intensity, and a mean heart rate (HR) of 95.6–120.5 beats per minute (bpm) during low-intensity exercise. The respective HRmax ranges were piloted in a separate sample of participants before the start of the study. In addition, HR was monitored during cycling to empirically align changes in heart rate with the corresponding exercise load (WATT). To this end, the participants' heart rates were observed using a chest strap connected to the cycling ergometer (one-lead ECG) to track changes in mean heart rate and cycling speed. As shown in Figure 4, the average HR during cycling fell within the range of those intensities. Cycling speed and exercise load were controlled by the ergo_win2003 software package (Daum Electronics).

During the warm-up and cool-down periods, totaling 4 min, participants were instructed to maintain an exercise load of 70 Watts, corresponding to 50%–63% of their maximum heart rate (HRmax). After the warm-up, the participants engaged in two periods of cycling at low to moderate intensity. Each period involved cycling at a constant load of either 85 Watts (low intensity) or 100 Watts (moderate intensity) for 5 or 7 min, respectively, reaching up to 76% of the participants' maximum heart rate (see Figure 1). The order of intensity (low vs. moderate) was counterbalanced across participants. Half of the participants followed a sequence of warm-up, low-intensity, moderate-intensity, and cool-down. The other half of the participants cycled in the order of warm-up, moderate intensity, low intensity, and cool-down. This design ensured that no specific cognitive subtasks during testing (described in the next section) were associated with a particular intensity level.

#### 2.2.3 Cognitive tasks

The cognitive tasks comprised the Verbal Learning Memory Test [VLMT; German version (39)] and the Regensburger Word Fluency Test [RWT; German version (40)]. The VLMT is the German version of the Auditory Verbal Learning Test (41). This test measures cognitive performance related to verbal learning and recall. The VLMT consists of two parts: the first involves immediate recall of a list of 15 German nouns, which participants must repeat across five trials. Between these trials, a distractor word list is presented to ensure the original list is not repeated. The second part of the VLMT assesses recall of the original word list. The lists (both to-be-remembered and distractor lists) are read aloud by an experimenter at a rate of one word per second. Participants are asked to recall as many words as possible. The cognitive functions assessed by the VLMT are controlled by prefrontal brain structures, the temporal lobe, and the hippocampus memory systems (42).

The RWT is a word fluency task. It assesses mental processing speed and cognitive functions linked to executive functions (controlled by prefrontal networks) such as the cognitive control of attention, working memory, and cognitive flexibility including cognitive switching (43, 44). It consists of four subtests and testing blocks, each lasting 2 min. During these test blocks, participants have to name as many words as possible that correspond to the given letter (for example, to create words beginning with the letter P) or that fit into a specified semantic category (for example, to create words from the category “fruits”). Switching between the testing blocks requires a letter or semantic category switch. Switches include letter and category switches that alternate according to a predefined rule (see Figure 1). The rules for the initial letters and semantic categories are described in the task manual (see Footnote)^1^.

Throughout both cognitive tests (VLMT and RWT), the participants provided their answers orally. Their responses were documented for later analysis. No feedback was given regarding their answers and performance. For both the VLMT and the RWT, parallel test versions were used in the EC and CC, respectively, to avoid conflating the task novelty and familiarity effects of interest with temporal-repetition effects.

#### 2.2.4 Task administration pre-post and during the exercise condition vs. control condition

The VLMT (39) was administered before and after the exercise condition (EC) or the control condition (CC). The first part of the VLMT was conducted immediately after the initial baseline measurement (baseline 1). In the EC, before the acute exercise bout (Figure 1). The delayed recall (the second part of the VLMT) took place after a 20-minute retention interval, following either the 16-minute acute bout of exercise (EC) or the control condition (CC).

The RWT (40) was conducted during the exercise condition (EC) or the control condition (CC). The testing included all four subtests (letter and semantic categories). The subtests were completed during the cycling exercise (EC) or while participants were seated on the ergometer without cycling (CC). A 1-minute break between the subtests was provided to facilitate the letter and semantic category switches (see Figure 1).

A total of three blocks of cognitive testing were conducted, which included both tasks (see Figure 1). The first cognitive testing (VLMT) was administered after baseline 1; participants remained seated on the cycling ergometer in the EC and CC conditions. The second block of cognitive testing occurred either during the acute bout of aerobic exercise (EC) or while sitting quietly on the cycling ergometer (CC) without exercising (RWT). The third cognitive testing block (recall of VLMT) was performed after the acute bout of exercise (EC) or after the CC. In both conditions, the participants remained seated on the ergometer.

### 2.3 Self-report

#### 2.3.1 Physical activity and current mood

The habitual physical activity level was evaluated using the Global Physical Activity Questionnaire [GPAQ; German version (38)]. The GPAQ assesses the regular physical activity over a week in three domains: a) commuting to and from work, b) traveling to and from various locations, and c) physical activity during leisure time each week. It assesses the intensity, duration, and frequency of physical activity within each domain. Additionally, participants completed the German version of the Positive and Negative Affect Schedule [PANAS (45),]. The PANAS measures positive and negative affect using 10 adjectives that describe emotional states experienced at the moment, rated on Likert scales. This allowed for the control of mood effects.

### 2.4 Psychophysiological measures and preprocessing

Changes in cardiac activity (mean heart rate) were recorded during the two baseline periods (resting state) and during the cognitive testing, which was conducted either while participants were exercising (EC) or while sitting on the bicycle (CC). Cardiac activity was recorded with the Bioradio system (Great Lakes NeuroTechnologies) and the BioCapture software (Great Lakes NeuroTechnologies). The Bioradio system is a mobile, wireless amplifier for recording biosignals. Heart rate can be recorded via a three-lead electrocardiogram and finger plethysmography, with maximum sampling rates of 4,000 Hz for the ECG and 1,500 Hz for plethysmography. Psychophysiological data, including ECG and finger plethysmography, were collected. Changes in heart rate were analyzed offline with the ARTiiFACT (46) software. The raw ECG data were sampled at 1,000 Hz. The signal amplitude was amplified by a factor of 100 to improve R-wave detection and ensure artifact-free R-R interbeat interval (IBI) detection. All datasets were manually inspected for artifacts, and any time intervals containing noisy data were excluded from further analysis. The rejected data segments were corrected using the cubic spline interpolation algorithm available in the ARTiiFACT software. The preprocessing and analysis followed the recommendations and guidelines implemented in the ARTiiFACT software. Additionally, resting heart rate variability (HRV) was analyzed during the two baseline periods. The mean RR values were used as outcome measures for changes in mean HR at rest. The high-frequency band (HF-HRV) was analyzed as a reliable indicator of HRV changes at rest associated with vagal control (46).

### 2.5 Data analysis

The data were statistically analyzed in a repeated-measures design with the within-subject factor “condition” (EC vs. CC). The factor “group” was included as a between-subjects factor to examine the influence of order-dependent selective effects of the testing time and context, i.e., whether the cycling exercise was performed on day 1, when the cognitive tasks were novel, or on day 2, when the cognitive tasks were already familiar. Cognitive performance in the verbal learning and memory task [VLMT (39)] was statistically compared after the experimental conditions (EC and CC, respectively) and across the testing times (day 1 vs. day 2). The outcome measures in the VLMT (39) comprised recall performance (the number of words correctly recalled from the original VLMT list) before and after the experimental sessions. In addition, the learning curve for the VLMT blocks performed before the exercise was analyzed in the EC and CC to control for context effects independent of the physical exercise.

The outcome measures of cognitive performance in the word fluency task [RWT (40)] included correct performance (accuracy) in the letter and the semantic category blocks (RWT letter, RWT letter switch, RWT semantic category, and RWT semantic category switch, respectively). The performance (accuracy) was compared between the experimental conditions (EC vs. CC) and between the testing sessions (whether the cycling exercise was carried out on day 1 or day 2).

Changes in mean heart rate were compared between the EC and CC conditions and between the testing sessions (day 1 vs. day 2). The analysis included changes in mean HR at rest [pre (baseline 1) to post (baseline 2)]. In addition, changes in mean heart rate were explored during the four two-minute blocks of letter- or semantic category-related word generation (see Figure 1) in the exercise condition (EC) and the control condition (CC).

Furthermore, changes in heart rate variability (HRV) were analyzed. Comparisons included comparisons between the two baseline conditions and between the EC and CC. The parameter HF-HRV (High-Frequency Heart Rate Variability) has proven to be the most reliable HRV marker for assessing vagally (parasympathetically) mediated cardiac control (for details, see sections 1, 4, and 5). Measured during rest, it serves as an individual's indicator of habitual ANS balance and degree of dominance of vagal activity at rest. Pre-post comparisons thus help to examine the short-term effects of the intervention (EC and CC) on resting vagal control, i.e., whether vagal control returned to baseline levels after cognitive testing with (EC) or without exercise (CC). Absolute values (HF-HRV in ms^2^) reflecting total parasympathetic activity were used as outcome measures.

Moreover, correlations were performed between the mean HR or HF-HRV parameter at baseline (baseline 1) and self-reported physical activity (GPAQ dimensions including physical activity during leisure time or during work) to determine the relationship between vagally-mediated cardiac activity at rest and the degree of habitual physical activity. In addition, correlation analyses were performed between physical activity (GPAQ dimensions), mean HR or HRV parameters, and cognitive performance in the VMTL or RWT to explore relationships among individual differences in physical activity, cardiac control at rest, and changes in mean HR during testing (EC vs. CC).

Where appropriate, statistical values, including F and *p*-values, are reported corrected for violations of sphericity using the Greenhouse-Geisser correction. For the results, effect sizes (*η*p², r) are reported, together with 95% CIs, for all repeated-measures designs and correlation analyses. For more than two *post hoc* comparisons, *p*-values are reported after correction using the Bonferroni-Holm procedure. In the exploratory correlation analyses, *p*-values are reported uncorrected.

## 3 Results

### 3.1 Sociodemographic data—descriptive data analytics

The participant sample comprised a homogeneous group of healthy adults with respect to age, sex, and sociodemographic background (see Table 1). Participants did not report any drug use other than alcohol, nicotine, or occasionally cannabis. Additionally, four women indicated they were using contraceptive methods. All participants had achieved at least university entrance qualifications and were students pursuing minor, major, or postgraduate degrees. Data from one participant who participated in only one of the two laboratory sessions were excluded. Consequently, the final sample consisted of 19 adults (10 women and 9 men) with a mean age of 24.32 years (*SD* = 2.91 years). Women and men did not differ significantly in age. Participants in the final sample reported experiencing more positive than negative affect (Table 1). According to the GPAQ questionnaire, none of the individuals indicated engaging in vigorous exercise related to their job or education. However, they reported engaging in moderate to vigorous physical activity during leisure time. Additionally, all participants reported sitting for at least 240 min (4 h) per day. Overall, the participants reported being physically active per day. For a detailed overview, see Table 1.

### 3.2 Cognitive performance before and after the acute bout of aerobic exercise

#### 3.2.1 Verbal learning and memory performance

The performance of the verbal learning memory test [VLMT (39)] followed a typical learning curve. There was a significant main effect of the factor “recall performance,” *F*(6, 102) = 92.78, *p* < 0.001, *partial eta squared* = 0.85, showing that recall performance improved across repeated list learning. There was no significant difference between the experimental conditions: the factor “experimental condition”, comparing the exercise (EC) and the control (CC) conditions, was not significant, *F*(1, 17) = 0.60, *p* = 0.46, *partial eta squared* = 0.33, and there was no significant interaction between the factors “recall performance” and “experimental condition”, *F*(6, 108) = 1.28, *p_GG_* = 0.27, *partial eta squared* = 0.066.

**Figure 2:**
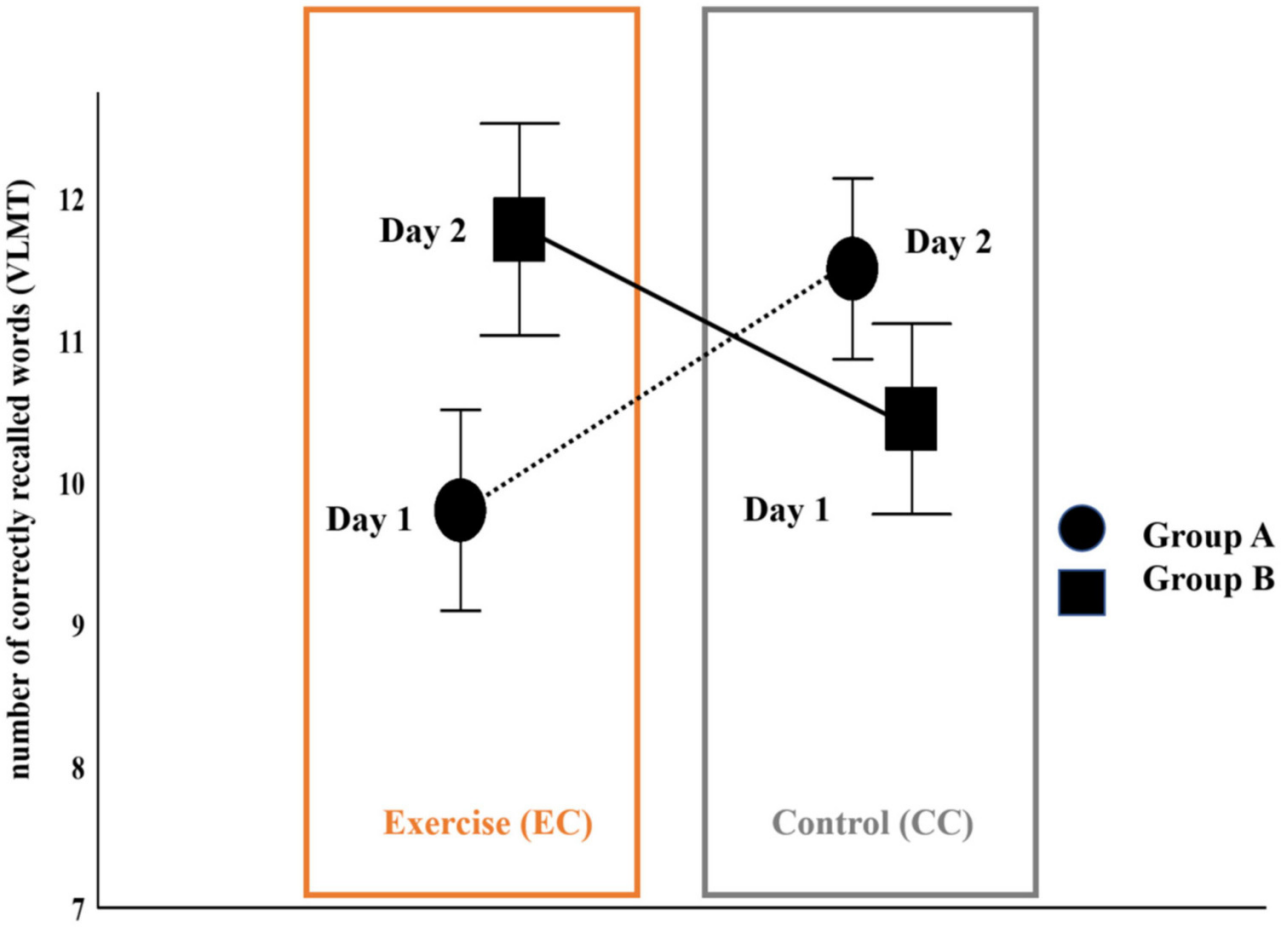
Cognitive performance in the VLMT across the testing time, depending on whether exercise was done on day 1 with the control (CC) on day 2 (group A) or vice versa (group B). Error bars represent standard errors. Means and CI (±95%) are plotted in Table 2 below the figure. For details, see Methods, Results, and Discussion.

The recall performance of the VLMT (39) that followed the intervention (EC or CC) showed no significant difference between the exercise condition (EC) and the control condition: the factor “experimental condition” was not significant, *F*(1, 17) = 0.27, *p* = 0.62, *partial eta squared* = 0.16. However, the factor “testing time” (exercise on day 1 or day 2/control condition on day 1 or day 2) significantly influenced the recall performance in the experimental conditions (EC vs. CC). This was demonstrated by the significant interaction between the factors “experimental condition” and “testing time”, *F*(1, 17) = 18.48, *p* < 0.001, *partial eta squared* = 0.52. Post-hoc comparisons yielded the following significant effect: participants who exercised on the first day showed better recall performance in the control condition on day 2, *p_corrected_* = .016. For an overview, see Figure 2.

**Table 2:** The table summarizes the effects illustrated in Figure 2, including means and confidence intervals.

Group	Exercise Condition	Day	Control Condition
Group A	Means & CI (±95%)		Means & CI (±95%)
Day 1	9.8 (8.31–11.29)	Day 2	11.5 (10.16–12.84)
Group B	Means & CI (±95%)		Means & CI (±95%)
Day 2	11.78 (10.20–13.35)	Day 1	10.44 (9.03–11.85)

### 3.3 Cognitive performance during the aerobic exercise

#### 3.3.1 Word fluency and cognitive switching

**Figure 3:**
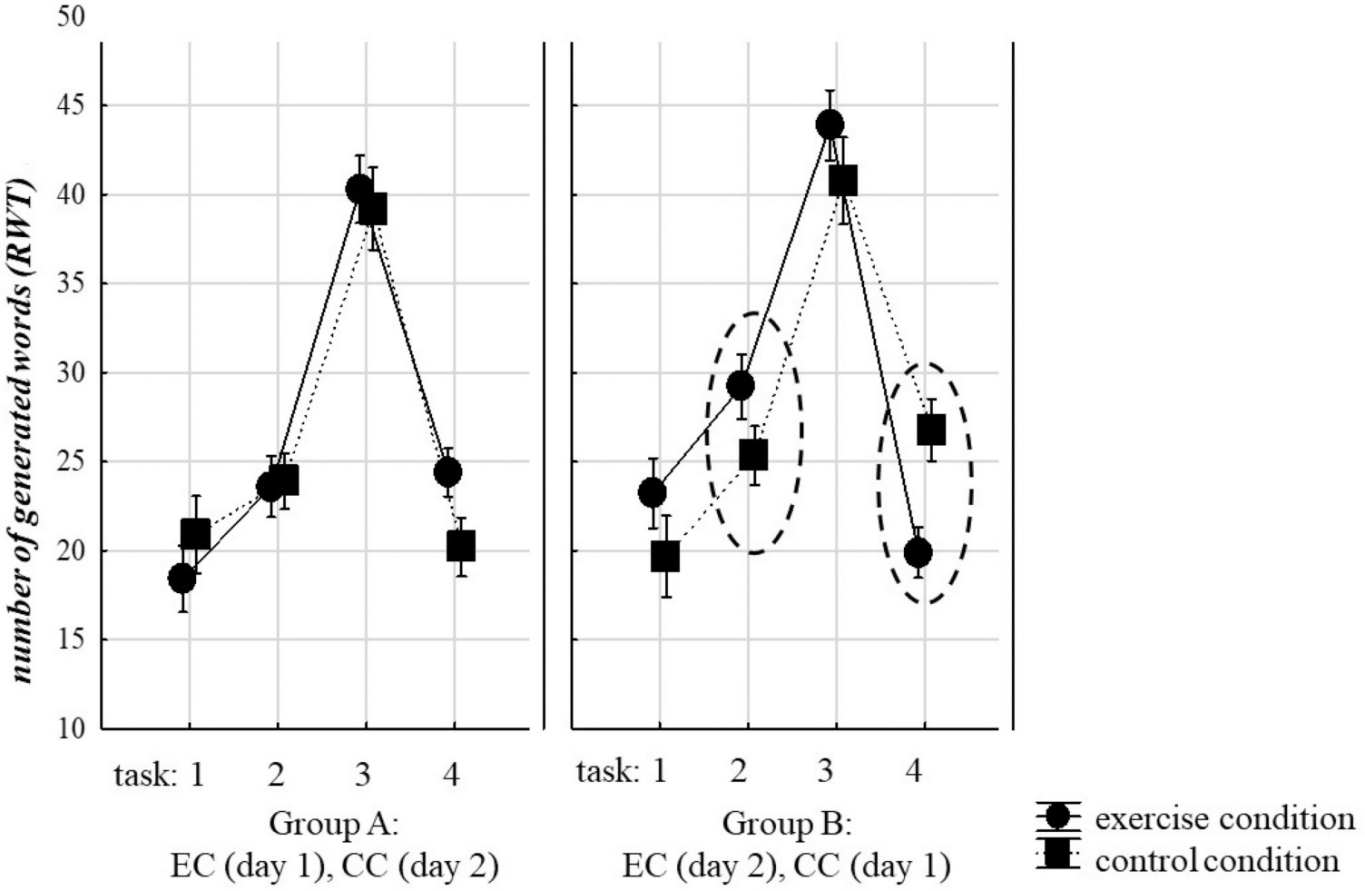
Cognitive performance in the word fluency test [RWT (40)] during exercise (EC) and control (CC) conditions and testing time [exercise on day 1 and control on day 2 (group A) or vice versa (group B)]. Significant effects are indicated by circles (see circles). Error bars: standard errors. Means and CI (±95%) are plotted in Table 3 below the figure. For details, see Methods, Results, and Discussion.

Performance in the RWT (40), which measures word fluency and cognitive switching, differed significantly between the four word generation subtests (letter vs. semantic categories), as shown by the significant main factor “performance”, *F*(3, 51) = 168.22, *p* < 0.001, *partial eta squared* = 0.91. As illustrated in Figure 3, the performance was best in the semantic category (subtest 3).

**Table 3:** The table summarizes the effects illustrated in Figure 3, including means and confidence intervals.

Group A	Test	Day 1	Exercise condition	Day 2	Control Condition
			Means & CI (±95%)		Means & CI (±95%)
Task 1	Letter 1		18.4 (14.48–22.17)		20.9 (16.33–25.47)
Task 2	Letter 2 switch		23–6 (19.96–27.24)		23.9 (20.57–27.23)
Task 3	Semantic 1		40.3 (36.31–44.29)		39.2 (34.32–44.08)
Task 4	Semantic 2 switch		24.4 (21.56–27.24)		39.2
Group B	Test	Day 2	Exercise Condition	Day 1	Control Condition
Task 1	Letter 1		23.2 (19.09–27.35)		19.6 (14.85–24.49)
Task 2	Letter 2 switch		29.2 (25.39–33.06)		25.3 (21.83–28.84)
Task 3	Semantic 1		43.8 (39.69–48.09)		40.7 (35.63–45.92)
Task 4	Semantic 2 switch		19.8 (16.90–22.84)		26.7 (23.09–30.46)

The performance on the four subtests did not differ significantly between the exercise condition (EC) and the control condition (CC). The main factor “experimental condition” was not significant, *F*(1,17) = 2.64, *p* = .12, *partial eta squared* = 0.13. Moreover, the main factor “performance” did not show a significant interaction with the factor “experimental condition” (EC and CC), *F*(3,51) = 1.46, *p* = .24, *partial eta squared* = 0.07, suggesting that the performance in each subtest did not differ significantly during exercising from the control condition of no exercise.

However, the factor “testing time” (exercise on day 1 or day 2/control condition on day 1 or day 2) significantly influenced the performance in the experimental conditions (EC vs. CC). This was demonstrated by the significant interaction between the factors “performance”, “experimental condition”, and “testing time”, *F*(3, 51) = 8.70, *p* < 0.001, *partial eta squared* = 0.34.

Post hoc comparison of this interaction effect revealed that cognitive performance in the second letter subtest (after the letter switch) was better in the exercise condition (EC) than in the control condition (CC) in those participants who had exercised on the second day of the testing, *p*_corrected_ = 0.056, whereas performance did not differ in the exercise and control condition in those participants who had exercised on the first day of the testing *p*_corrected_ > .1. In contrast, the cognitive performance in the subtest 4, which comprised the second semantic category (after the category switch) was significantly reduced in the exercise condition (EC) compared to the control condition (CC) in those participants who had exercised on the second day of the testing, *p*_corrected_ = .008, whereas performance in the same subtext did not significantly differ between the exercise condition (EC) and the control condition (CC) in those participants who had exercised on the first day of the testing *p*_corrected_ > .1, see Figure 3.

### 3.4 Psychophysiological effects of the acute bout of aerobic exercise

#### 3.4.1 Pre-Post

Changes in mean heart rate (mean HR) assessed during rest at the two baseline conditions before and after the intervention differed significantly. The main factor “pre-post” was significant, *F*(1,17) = 31.0, *p* < 0.0001, *partial eta squared* = 0.68. Furthermore, changes in mean heart rate (mean HR) differed across experimental conditions (EC vs. CC). The factor “experimental condition” showed a significant effect, *F*(1,17) = 6.5, *p* = 0.022, *partial eta squared* = 0.28. In addition, mean heart rate differed as a function of the interaction of the factors “pre-post” and “experimental condition”, *F*(1,17) = 15.50, *p* < 0.001, *partial eta squared* = 0.48. The analysis of this significant interaction showed that mean HR was significantly higher after (baseline 2) than before the acute bout of exercise (baseline 1). Post-hoc tests showed that this was significant in the exercise condition, *p*_corrected_ < .001. In the control condition, mean heart rate did not differ pre- to post-intervention, *p*_corrected_ *p* > .6.

**Figure 4:**
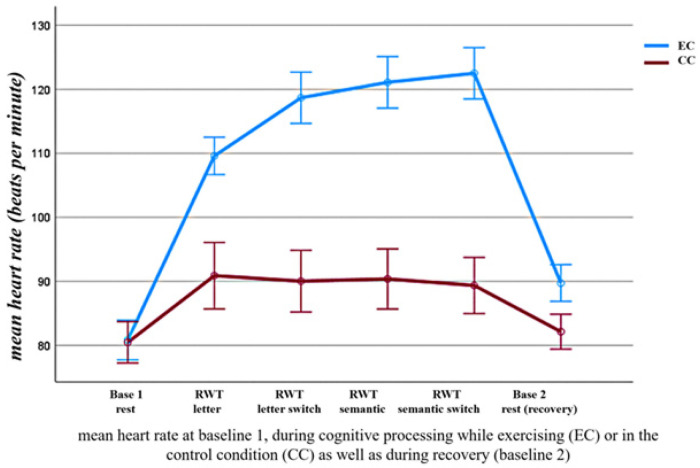
Changes in mean heart rate (mean HR) during the word fluency test [RWT (40)] in the exercise condition (EC) and control condition (CC). Base: Baseline; Cond: condition; EC: exercise; CC: control. Means and standard errors. Means and CI (±95%) are plotted in Table 4 below the figure. Significant comparisons (a/b, c/d) are indicated with an *. See Methods and Results for details.

The factor “testing time” did not significantly influence these changes in mean heart rate. There was no significant interaction of the factors “testing time”, “experimental condition”, and “pre-post”, *F*(1,17) = 0.13, *p* = 0.72, *partial eta squared* = 0.01. In addition, mean heart rate (mean HR) did not differ between the two experimental conditions (EC and CC) before the intervention (at baseline 1), *p* > .05, see also Figure 4, Tables 4, 5.

**Table 4:** The table summarizes the effects illustrated in Figure 4, including means and confidence intervals.

Experimental Condition (EC/CC)	Task condition	Mean	CI ±95%
EC	RWT-letter^a^	109.52	103.21–115.83
EC	RWT-switch^b^	118.63	109.94–127.33
EC	RWT-semantic	121.05	112.28–129.81
EC	RWT-switch	122.46	113.75–131.18
CC	RWT-letter	90.96	79.68–102.22
CC	RWT-switch	90.067	79.60–100.53
CC	RWT-semantic	90.38	80.17–100.60
CC	RWT-switch	89.35	79.81–98.90

Significant comparisons are indicated with a letter.

**Table 5:** Changes in mean HR and vagally mediated HF-HRV during rest, pre to post, exercise (EC) or control (CC) conditions. HF-HRV in absolute units (ms^2^). Significant effects are marked with superscripts; significant comparisons (a/b, c/d) are indicated with an *: means, standard deviations, and CI (±95%)**.** See Methods and Results for details.

Change in resting state (mean HR and vagally mediated HF-HRV, pre to post, during EC or CC)	Mean	Standard Deviation (SD) and CI (±95%)
a: EC: resting HR (meanHR) pre	80.99*^b^	13.52 (74.39–87.59)
b: EC: resting HR (meanHR) post	89.79*^a^	12.50 (83.58–96.00)
c: CC: resting HR (meanHR) pre	80.51	14.05 (73.51–87.51)
d: CC: resting HR (meanHR) post	82.13	11.88 (76.20–88.06)
e: EC: HF-HRV (vagal modulation) rest pre	363.92*^c^	366.37 (181.21–546.64)
f: EC: HF-HRV (vagal modulation) rest post	119.41*^d^	82.81 (78.44–160.38)
g: CC: HF-HRV (vagal modulation) rest pre	353.97	383.06 (164.85–543.09)
h: CC: HF-HRV (vagal modulation) rest post	258.55	270.64 (126.84–390.26)

Similar to the changes in mean HR, changes in the vagally mediated HRV, as indicated by the high-frequency band HF-HRV, differed significantly between the two baseline conditions. The factor “pre-post” was significant, *F*(1, 17) = 8.33, *p* = 0.01, *partial eta squared* = 0.32. The changes differed between the experimental conditions (EC vs. CC) from pre- to post. This was indicated by the significant interaction of the factors “pre-post” and “experimental condition”, *F*(1, 17) = 4.33, *p* = 0.05, *partial eta squared* = 0.19. The vagally mediated HF-HRV activity was lower during the second baseline assessment (baseline 2) than during baseline 1 in the exercise condition (*p*_corrected_ < .001) and compared to the control condition at baseline 2 (*p*_corrected_ = .053). For an overview, see Tables 4, 5. There was no significant interaction with the testing time (day 1 or day 2).

#### 3.4.2 During the conditions (EC and CC)

Analysis of changes in mean heart rate (mean HR) during the intervention revealed significant differences across the four cognitive subtests. The factor “cognitive testing” was significant, *F*(3, 51) = 18.36, *p* < .001, *partial eta squared* = 0.51. The changes in mean heart rate differed significantly across experimental conditions, as indicated by a significant interaction between the factors “cognitive testing” and “experimental condition”, *F*(3, 51) = 11.24, *p* < .001, *partial eta squared* = 0.39. As illustrated in Figure 4, in the control condition (CC), the mean heart rate did not change significantly during cognitive testing. However, it was significantly higher during cognitive testing when individuals were exercising. In addition, when simultaneously exercising (EC), changes in mean heart rate increased from the first to the second cognitive performance block of testing (*p*_corrected_ < .05) and reached a plateau with no further change across the sequential testing blocks (all *p*_orrected_ > .05; see Figure 4). There was no significant interaction with the testing time (day 1 vs. day 2). The interaction between the factors “cognitive testing”, “experimental condition”, and “testing time” was not significant, *F*(3, 51) = 0.22, *p* = .88, *partial eta squared* = 0.01.

### 3.5 Correlation analyses—exploratory analysis

#### 3.5.1 Cognitive performance and HRV at rest

Exploratory correlation analyses between cognitive performance measures [VLMT (39) and RWT (40)] in both the exercise and control conditions and vagally mediated HF-HRV at rest during the first baseline condition (baseline 1) were significantly correlated with cognitive performance in the RWT in both experimental conditions, i.e., during exercise (EC) and while sitting still without cycling (CC). Significant negative correlations of moderate effect size were found. This included the correlations between HF-HRV and cognitive performance in the first two blocks of the RWT, which comprised letter-related category generation and letter-related category switching (see Table 6). No correlations were found between HF-HRV at rest and cognitive performance in the VMLT (39), which was examined pre- to post-exercise, nor between pre- and post-exercise HF-HRV (all *p*_uncorrected_ > .05).

**Table 6:** Table 6 shows correlations between vagally mediated HF-HRV at rest and performance in the RWT word fluency subtests during exercise (EC) or no exercise (CC). Correlations (Pearson's r), exploratory testing, *p* uncorrected. See Methods and Results for details.

	RWT_letter_ EC	RWT_letter switch_ EC	RWT_letter_ CC	RWT_letter switch_ CC
HF-HRV_rest_	*r* = −0.542, *p*_uncorrected_ = 0.021	*r* = −0.588, *p*_uncorrected_ = 0.01	*r* = −0.489, *p*_uncorrected_ = 0.04	*r* = −0.523, *p*_uncorrected_ = 0.03

#### 3.5.2 Cognitive performance, habitual physical activity, heart rate, and HRV

Exploratory correlation analyses between the self-reported regular physical activity (GPAQ) of the participants in the domains of work, transport or leisure time did not reveal significant correlations with cognitive performance in the VMLT (39) or RWT (40) (all *p*_uncorrected_ > .05). However, self-reported regular vigorous activity during leisure time was significantly negatively correlated with the mean changes in mean heart rate during almost all cognitive testing blocks while exercising (EC: RWT_letter:_ *r* = −0.49, p_uncorrected_ = 0.03; RWT_letterswitch:_ *r* = −0.53, *p*_uncorrected_ = 0.02, RWT_semantic:_ *r* = −0.46, *p*_uncorrected_ = 0.05, RWT_semanticswitch_: *r* = −0.43, *p*_uncorrected_ = 0.07), but not in the control condition of cognitive testing without exercise (CC: all *p*_uncorrected_ > .05). During rest, cardiac activity (mean HR, mean RR, or mean HF-HRV) assessed during the first baseline condition (baseline 1) did not significantly correlate with GPAQ scores (38), including physical activity (sum score) or physical activity during leisure time (all *p*_uncorrected_ > .07).

## 4 Discussion

This study investigated changes in cognitive performance induced by an acute bout of aerobic exercise in healthy adults, as an important target population for health prevention. Following previous studies and the research questions discussed in the introduction, the study explored whether cognitive performance is improved when exercise sessions are short (in total 16 min), include cycling at low to moderate intensity, and cognitive performance is assessed during, before and after the exercise, as well as across the testing sessions to examine order-dependent selective effects of the exercise intervention when the cognitive tasks are novel or already familiar to the participants. Cognitive performance was examined in two standardized cognitive tasks of verbal memory and verbal fluency to investigate the specific effects of exercise in these cognitive domains.

The main findings suggest that cycling at low to moderate intensity for a short duration, lasting 16 min, can enhance cognitive performance. The effects, however, are modulated by order-dependent selective effects of the testing time and the specific tasks involved. Therefore, for aerobic exercises of low to moderate intensity and short duration, the present study supports the notion that effects on cognitive processing depend on which type of cognitive processing is assessed, when it is assessed (before, during or after the exercise), and whether the exercise is performed on day 1 for new cognitive tasks or on a second cognitive testing when the cognitive tasks are already familiar.

### 4.1 Cognitive performance pre- to post-exercise: effects of order-dependent selective effects of the testing time

Verbal learning memory was assessed with the VLMT (39) before and after the two experimental conditions [exercise condition (EC) and the control condition of no exercise (CC)], see Figure 1. The memory recall followed the word list learning blocks after a 20-minute retention interval, and 4 min after recovery from either the exercise (EC) or the control condition (CC). In both experimental conditions, the participants were sitting comfortably on the ergometer in the same body position. The VLMT (39) allows a standardized assessment of learning and memory recall after cognitive interference. In other words, it assesses immediate and delayed retrieval of semantic information after competing semantic memory content from distractor lists. Therefore, successful retrieval in the VLMT (39) requires different cognitive processes. These processes may include top-down attention, inhibitory control, working memory, semantic processing, and goal-directed retrieval of previously learned and stored information (42, 47).

Previous studies have explored whether acute bouts of aerobic exercise affect the retrieval of semantic information in memory tests [for an overview, see (14, 26, 48)]. Only a few of the exercise studies examined recall performance following cognitive interference, such as in the VLMT (39). Some of these studies suggest that aerobic exercise can improve memory performance by reducing cognitive interference elicited by distracting information [(49, 50), for a discussion (51)]. In addition, some studies suggest that specifically, the ability to retain and switch between multiple memory representations benefits from low to moderate intensity aerobic exercise both during and after exercising (52).

However, not all studies have supported these findings. Moreover, the benefits of memory retrieval from an acute bout of exercise may extend beyond the immediate retention or exercise recovery period. Studies that gathered data days after the exercise session showed improved recall after several days. However, this enhancement was observed only when memory was retested on these days following another bout of exercise (50).

The current findings can enhance the understanding of these earlier observations. In the present study, recall accuracy (i.e., the number of correctly remembered words) in the VLMT (39) was compared between the two experimental conditions of exercise and no exercise (control condition) and as a function of the order-dependent selective effects of the testing time (day 1 or day 2). Recall was not significantly improved or worsened by the 16 min of low-to-moderate intensity aerobic exercise, as measured after the cycling exercise, compared with pre-post exercise and the control condition. The within-subject crossover design allowed for conducting interindividual (between) and intraindividual (within) comparisons, showing the reasons for this insignificant effect. The analyses revealed that the timing of the exercise (day 1 or day 2) was important. The results presented in Figure 2 illustrate these order-dependent selective effects of the testing time: the acute bout of cycling exercise influenced recall performance differently based on whether the exercise was carried out on day 1, when the task was novel, or on day 2, when the task was already familiar: intra-individual within-subject comparisons suggested that participants who exercised on day 1 could improve their memory performance in the control condition on day 2, when the task was familiar; see Figure 2. In the control condition of no aerobic exercise, no significant differences in recall performance were observed between day 1 and day 2. Therefore, the positive effects of exercising on the memory performance in the VLMT (39) cannot be due to carry-over effects from repeated testing.

### 4.2 Cognitive performance during the exercise: effects of order-dependent selective effects of the testing time

Order-dependent selective effects of the testing time also affected how the acute bout of cycling exercise influenced cognitive performance during the RWT (40), which was conducted while participants were cycling. The subtests of the RWT (40) comprise word generation according to phonetic cues (words starting with a particular letter) or semantic categories (words belonging to a particular semantic category). The participants generate the words and name them in a certain amount of time. Therefore, word fluency tests such as the RWT (40) are frequently used in the literature to test language processing and executive cognitive functions (52, 53). The letter category subtests examine cognitive control of attention, working memory, semantic processing, cognitive flexibility including cognitive switching, and cognitive processing speed. Performance in the semantic category subtests additionally requires semantic knowledge. Switching the categories from letter to letter, from letter to semantic categories, and between semantic categories requires cognitive control and the inhibition of competitive stimulus associations to prevent perseveration errors (52, 53).

The cycling exercise enhanced word fluency in the RWT (40). Compared to the control condition of no exercise (CC), the acute bout of cycling exercise (EC) improved cognitive performance during the second block of letter-based word generation after letter-based task switching within the first 5 min of the aerobic exercise. This enhancement effect was observed only in participants who exercised on the second day, when the cognitive task was no longer novel. Interestingly, the performance in the second semantic category subtest conducted after semantic task switching during the last five minutes of the cycling exercise exhibited a different testing effect. During this period, performance was reduced in the participants who exercised on day 2 compared to day 1 when they were tested without cycling (see Figure 3).

Therefore, similar to the results for memory performance after the exercise, the results during the exercise support the idea that order-dependent selective effects influence the effects of exercise on cognitive processing and cannot be interpreted independently of it.

Moreover, the results suggest several interrelated effects between cognitive processing and short bouts of exercise, the role of arousal, and individual factors in these processes, as discussed next.

### 4.3 Role of arousal, neurophysiological mechanisms

Previous aerobic exercise studies indicate that maintaining an optimal level of arousal during the aerobic exercise is important for learning, consolidation, and retrieval of information (18, 20). This optimal arousal level may be characterized by an inverted-U relationship between arousal and performance, as proposed by the Yerkes-Dodson law (18, 54). However, what constitutes this optimal arousal level can depend on several factors (18, 20, 55–58). This can include the type of cognitive task (such as perceptual, motor, semantic, or episodic memory), the nature of the performance (whether speed or accuracy is prioritized), the difficulty of the task, the intensity of the exercise, carried out at a steady state or until exhaustion (21, 22). Similarly, the context in which the information is learned, consolidated, and retrieved matters.

Changes in mean heart rate (mean HR) examined pre- and post-exercise and during cognitive testing can serve as a proxy for the degree of arousal induced by the tasks in the control and exercise conditions. This revealed significantly greater changes in mean heart rate in the exercise condition (EC) across all blocks of the RWT (40) than in the cognitive processing condition (CC) without exercise, as shown in Figure 4.

In addition, mean heart rate remained elevated after exercise. It was significantly higher during the second baseline measurement (post-intervention) in the exercise condition (EC) compared with the control condition (CC). In the control condition (CC), mean heart rate returned to baseline after cognitive testing and showed no significant difference between baseline 1 (pre-intervention) and baseline 2 (post-intervention; see Figure 4). This suggests that cycling at low to moderate intensity induced higher arousal than cognitive testing alone (without cycling in the CC). This increase occurred during the exercise (with a plateau reached after five minutes, see Figure 4) and remained elevated after exercise, when physiological arousal had already returned to baseline levels in the control condition (CC). This supports previous studies showing that exercise can interact with physical recovery after cognitive testing, even minutes after the testing period (50).

This is also suggested by changes in the vagally mediated HF-HRV, which indicate the degree of parasympathetic dominance in heart rate control, typically observed during rest (59). HF-HRV was significantly lower after cognitive testing in the exercise condition (EC) than before testing (baseline 1) and in the control condition (CC), in which vagally mediated HF-HRV was equally pronounced post-testing as pre-testing. Both mean HR and vagally mediated HF-HRV did not significantly differ between the exercise (EC) and control condition (CC) before the intervention (at baseline 1). Taken together, this suggests that the increase in physiological arousal during exercise and cognitive testing was accompanied by a withdrawal of parasympathetic control over heart rate during the recovery period, which coincided with the memory testing. However, the suggestions and interpretations of the present findings require further examination and validation in future studies due to several limitations of the present study (see the next section for details).

Neuroscientific theories of exercise-cognition interactions suggest that acute bouts of exercise can modulate activity in several brain networks. These include prefrontal and fronto-parietal attention and cognitive control networks, as well as memory networks (60). In addition, it has been suggested that exercising can decrease neural activity in brain structures not used during physical activity, as proposed by the theory of transient frontal hypofrontality (61). It has been proposed that a high physiological arousal level, or a combination of high arousal and hypofrontality, can negatively impact cognitive performance, especially at longer exercise durations and high exercise intensities until exhaustion (62). Thus, speculatively though, neither too low nor too high arousal, nor hypofrontality, nor a combination of these factors—such as exercising to the point of exhaustion—appear to have influenced the current findings. For aerobic cycling at low to moderate intensity and short duration, the present study suggests that additional factors beyond arousal effects should be considered.

### 4.4 Role of interindividual differences and HRV at rest—exploratory analyses

In the exercise condition (EC), the physiological arousal induced by the exercise differed with participants' self-reported physical activity levels and heart rate variability at rest. Leisure-time physical activity, as assessed with the GPAQ questionnaire (38), was negatively correlated with the increase in physiological arousal during the cognitive testing in the exercise condition (EC). This suggests that regular physical activity of moderate to high intensity during leisure time reduces the degree of physiological arousal induced by an acute bout of exercise during cognitive testing, but does not reduce the arousal caused by the cognitive task itself. Moreover, leisure-time physical activity did not correlate with any of the cognitive outcome measures. Interestingly, HF-HRV at rest (baseline 1) was negatively correlated with cognitive performance. This was observed in the letter-based word fluency tests during both the exercise condition (EC) and the control condition (CC). The letter-based word fluency subtests specifically require the suppression of words with shared linguistic associations and thus, cognitive inhibition (63).

HF-HRV measured at rest is a reliable measure of the parasympathetic, vagal modulation of cardiac activity and has been proposed as a proxy of a person's self-regulatory adaptability and neurovisceral integration (32, 35–37). Neurovisceral integration describes one of the fundamental mechanisms of mind‒body or body‒mind interactions, specifically the control of internal bodily signals (such as heart rate) by brain regions of the central autonomic network (CAN) (32, 64, 65). As recently reviewed, most previous research exploring the interaction between HRV at rest and cognitive processing, primarily among healthy adults, has revealed positive relationships between cognitive performance and HF-HRV in tasks requiring cognitive inhibition or affect regulation, but not in memory tasks (66).

Thus, although speculative, one explanation for the negative correlation between HF-HRV and the performance in the speeded letter-based word fluency tests is that, in healthy adults, greater vagally controlled self-regulation mediated by the CAN network (32) is associated with better cognitive inhibition that slows down the speed of processing in favor of accuracy. Another explanation is that higher vagal modulation of the heart rate at rest indicates lower physiological arousal and higher relaxation. Low arousal and relaxation may especially influence the speed of cognitive processing in the first minutes of cognitive testing. As stated in the Method section, the correlational results are exploratory, should be interpreted with caution, and require further validation in future research.

## 5 Conclusions: strengths, limitations, and outlook

This study adds to a still underrepresented literature on exercise-cognition interactions in low-intensity, short-duration aerobic exercise, and on cognitive domain specificity and order-dependent selective effects of testing time. The present study's approach examined cognitive processing before, during and after an acute bout of exercise and explored effects across testing sessions. This revealed that, for aerobic exercises of low-to-moderate intensity with a maximum duration of 16 min, exercise effects depend on order-dependent selective effects, whether the exercise is carried out within a novel or a familiar task context. The joint assessment of psychophysiological and cognitive outcome variables, along with interindividual differences, showed that examining different response levels (psychophysiological and cognitive) can be important for understanding exercise-cognition effects. Moreover, the present results encourage further research into resting-state measures, such as heart rate variability (HRV). The present results are only exploratory, but further investigations will help determine the relationships among exercise, its physiological effects, and those of regular physical activity, as well as cognitive processing, and the role of individual state and trait markers of self-regulation and cardiac neurovisceral control.

The present results revealed several interesting findings regarding the conditions under which a short bout of aerobic exercise can influence cognitive processing among healthy adults. Methodologically, the present study's strength lies in its multimethod design and focus on healthy young adults of similar age and occupational backgrounds. On the other hand, the results of the present study have limitations: First, the small sample size of the interindividual group comparisons. The small sample size prevents generalizing the effects to the broader population of young adults, increasing the Type II error risks in non-significant results and Type I inflation due to multiple testing. According to power estimates based on the range of effect sizes reported in the manuscript, the factorial designs would ideally have required a total sample size of about *N* = 34–74 participants to achieve the lowest partial etas and avoid type II errors and failing to detect a real effect. In contrast, for higher values, a total sample size of *N* = 10–18 would be recommended. Therefore, the results require validation in larger samples in future studies to increase power, avoid Type II errors, and avoid failing to detect a real effect, as well as preregistration. Regarding exercise intensity, control via WATT and heart rate was performed; however, future studies should include additional tests and measures to ensure normalization of exercise intensity. The same holds for HRV assessment, especially if one wants to use it as a measure of vagally controlled control during exercise. To this end, respiration should be controlled, as HRV and respiration, as well as vagal control, are closely related (67). Moreover, speech–respiration confounds should be well controlled to avoid potential impact on RWT performance. Importantly, future research should follow up on the order-dependent selective effects to clarify their mechanisms, which may relate to learning or familiarization effects across sessions. Cognitive, motor, and respiratory interference effects have been discussed recently (68). Research indicates that young healthy adults are a vulnerable target group at risk for mental health conditions (5, 69). This includes stress vulnerability and risk of depression or anxiety, which can negatively affect physical and mental health and may affect a significant proportion of young adults of working age. Previous research has not yet provided conclusive evidence on the optimal exercise intensity and the minimal duration required to elicit beneficial effects on different types of cognition among healthy adults [recent discussions (12–15)]. To ensure the successful transfer of findings to practical applications, further research is needed. This research should take into account the current findings regarding task and session effects to replicate these results. The goal is to make firm recommendations on how single-session, short-bout, low-to-moderate-intensity aerobic exercise protocols should be designed to positively influence the psychological, cognitive, and emotional well-being of young adults.

Once sufficient evidence is gathered, it will be possible to decide how to integrate short exercise sessions into young adults' daily routines, for example, through short activity breaks. Recently, suggestions have been made on how this could be achieved (29, 30, 70). Based on the findings and suggestions of meta-analytic studies, as well as the limitations noted in earlier research, these recommendations emphasize the need to discuss the cognitive benefits of short exercise sessions in a targeted manner to support cognitive health in adults [for an overview (23, 29, 70)]. The current study and its findings can enhance these recommendations as detailed above and summarized in Table 7. However, future research with similar designs and methods is necessary to validate and test these recommendations on larger samples. This will help achieve the goals.

**Table 7:** Summary of the results regarding how 16-minute cycling impacted cognitive performance in this study. These findings may inform exercise recommendations; please refer to the Conclusions section for details.

Summary of the results of the crossover study design Sample: Healthy young adults	
Testing time	
First day (exercise)	Second day (exercise)
The task is novel	The task is familiar
Cycling for 16 min at low to moderate intensity….	
no effect	enhances correct memory recall in the VLMT (39), when tested without exercising, provided the cognitive task was learned in combination with the exercise on day 1
no effect	improves switching of letter categories and reduces switching of semantic categories in word fluency in the RWT (40) compared to testing without exercise
…. enhances physiological arousal states during cognitive testing	
Influence of individual factors … (exploratory)	
regular vigorous physical activity during leisure time is negatively correlated with physiological arousal states during cognitive testing while exercising	
vagally mediated heart rate variability (HRV) at rest, as a proxy for cardiac self-regulatory capacity, is negatively correlated with word fluency and cognitive switching (letter categories) performance in the RWT (40), both during control (no exercise) and while exercising	

## Data Availability

The raw data supporting the conclusions of this article will be made available by the authors, without undue reservation.

## Footnotes

^1^RWT: The words had to be related to a given semantic category of a specific initial letter combination. In addition, the generated words had to originate in German, begin with different word stems, and not be proper nouns. Version A: letter: “P” words, letter change: “G” and “R” words; semantic category: “animals”, semantic category change: “sports” and “fruits”. Version B: letter: “M” words, letter change: “H” words and “T” words; semantic category: “food”, semantic category change: “clothes” and “flowers”.
